# Quantitative Analysis and Comparison Study of [^18^F]AlF-NOTA-PRGD2, [^18^F]FPPRGD2 and [^68^Ga]Ga-NOTA-PRGD2 Using a Reference Tissue Model

**DOI:** 10.1371/journal.pone.0037506

**Published:** 2012-05-18

**Authors:** Ning Guo, Lixin Lang, Weihua Li, Dale O. Kiesewetter, Haokao Gao, Gang Niu, Qingguo Xie, Xiaoyuan Chen

**Affiliations:** 1 Laboratory of Molecular Imaging and Nanomedicine, National Institute of Biomedical Imaging and Bioengineering, National Institutes of Health, Bethesda, Maryland, United States of America; 2 Department of Biomedical Engineering, Huazhong University of Science and Technology, Wuhan, Hubei, China; 3 Wuhan National Laboratory for Optoelectronics, Wuhan, Hubei, China; 4 Center for Molecular Imaging and Translational Medicine, School of Public Health, Xiamen University, Xiamen, China; University of Texas, M.D. Anderson Cancer Center, United States of America

## Abstract

With favorable pharmacokinetics and binding affinity for α_v_β_3_ integrin, ^18^F-labeled dimeric cyclic RGD peptide ([^18^F]FPPRGD2) has been intensively used as a PET imaging probe for lesion detection and therapy response monitoring. A recently introduced kit formulation method, which uses an ^18^F-fluoride-aluminum complex labeled RGD tracer ([^18^F]AlF-NOTA-PRGD2), provides a strategy for simplifying the labeling procedure to facilitate clinical translation. Meanwhile, an easy-to-prepare ^68^Ga-labeled NOTA-PRGD2 has also been reported to have promising properties for imaging integrin α_v_β_3_. The purpose of this study is to quantitatively compare the pharmacokinetic parameters of [^18^F]FPPRGD2, [^18^F]AlF-NOTA-PRGD2, and [^68^Ga]Ga-NOTA-PRGD2. U87MG tumor-bearing mice underwent 60-min dynamic PET scans following the injection of three tracers. Kinetic parameters were calculated using Logan graphical analysis with reference tissue. Parametric maps were generated using voxel-level modeling. All three compounds showed high binding potential (Bp_ND_ = k_3_/k_4_) in tumor voxels. [^18^F]AlF-NOTA-PRGD2 showed comparable Bp_ND_ value (3.75±0.65) with those of [^18^F]FPPRGD2 (3.39±0.84) and [^68^Ga]Ga-NOTA-PRGD2 (3.09±0.21) (p>0.05). Little difference was found in volume of distribution (V_T_) among these three RGD tracers in tumor, liver and muscle. Parametric maps showed similar kinetic parameters for all three tracers. We also demonstrated that the impact of non-specific binding could be eliminated in the kinetic analysis. Consequently, kinetic parameter estimation showed more comparable results among groups than static image analysis. In conclusion, [^18^F]AlF-NOTA-PRGD2 and [^68^Ga]Ga-NOTA-PRGD2 have comparable pharmacokinetics and quantitative parameters compared to those of [^18^F]FPPRGD2. Despite the apparent difference in tumor uptake (%ID/g determined from static images) and clearance pattern, the actual specific binding component extrapolated from kinetic modeling appears to be comparable for all three dimeric RGD tracers.

## Introduction

Members of integrin family play an important role in the regulation of cellular activation, survival and migration. Integrin facilitates the vascular cell proliferation which is necessary for tumor growth and metastasis [1]–[4]. Among the integrin receptor subtypes, α_v_β_3_ is one of the most important members because of its involvement in tumor angiogenesis and metastasis. Therefore, quantification of tumor integrin α_v_β_3_ level by non-invasive PET imaging has become an important tool for tumor diagnosis and treatment monitoring in both pre-clinical and clinical studies [5]–[8]. Great efforts have been made in developing radiolabeled integrin targeting agents [6], [9]–[12]. A variety of arginine-glycine-aspartic (RGD)-based probes have been made and investigated, since the cyclic RGD containing peptides have high affinity and selectivity for integrin α_v_β_3_. It has also been well documented that dimeric and multimeric RGD peptides are superior to the monomeric analogs [9], [13], most likely due to the polyvalency effect. RGD peptides have been radiolabeled and evaluated with ^18^F [14], ^64^Cu [15], ^68^Ga [16], [17], ^76^Br [12] and ^89^Zr [18] for integrin α_v_β_3_ targeted PET imaging. A number of ^18^F-labeled RGD peptide tracers have been tested in oncologic patients, including [^18^F]galacto-RGD [6], [^18^F]AH11585 [19] and [^18^F]FPPRGD2 [20]. However, all these compounds suffer from multistep time consuming and low yield synthetic procedures, limiting their widespread use as routine tracers in the clinic.

Recently, major advances have been made in simplifying ^18^F-labeled bioactive molecules [21]–[24]. The fluorophilic nature of aluminum is most attractive since it affords direct aqueous ^18^F-labeling by the formation of stable aluminum fluoride chelates. Previously we have successfully synthesized [^18^F]AlF-NOTA-PRGD2 through a kit formulation without the need of HPLC purification. The tracer showed high specific activity and has been tested in both xenograft [25] and myocardial infarct [26] models. NOTA-RGD conjugates have also been labeled with ^68^Ga, a generator based PET radionuclide [16], [17].

Visual inspection and simple standardized uptake value (SUV) assessment are insufficient to properly analyze the data acquired from a variety of tracers with different kinetic properties [27]. Moreover, when used for the evaluation of pharmacokinetic properties of a drug or tracer, quantitative data analysis from dynamic imaging can provide necessary parameters such as peak time, clearance rate, binding potential and volumes of distribution. Kinetic modeling with graphical analysis provides a visual way to distinguish different types of tracer accumulation in the initial studies of new ligands. Kinetic modeling is reliable and independent on scan duration and plasma clearance, and therefore is considered to be more favorable than SUV in the data analysis.

Logan graphical analysis is a widely accepted method for reversible tracer kinetic analysis [28]. It provides an estimate of the Distribution Volume, DV, by a simple plot without the necessity of specifying a particular tissue model [29]. It also shows marked advantages for generating kinetic parametric images because of its fast computation speed and robust performance over the high level noise in the time-activity curves of individual PET image voxels. Consequently, graphical analysis (GA) methods are particularly suitable for characterizing the kinetics of a new tracer.

**Figure 1:**
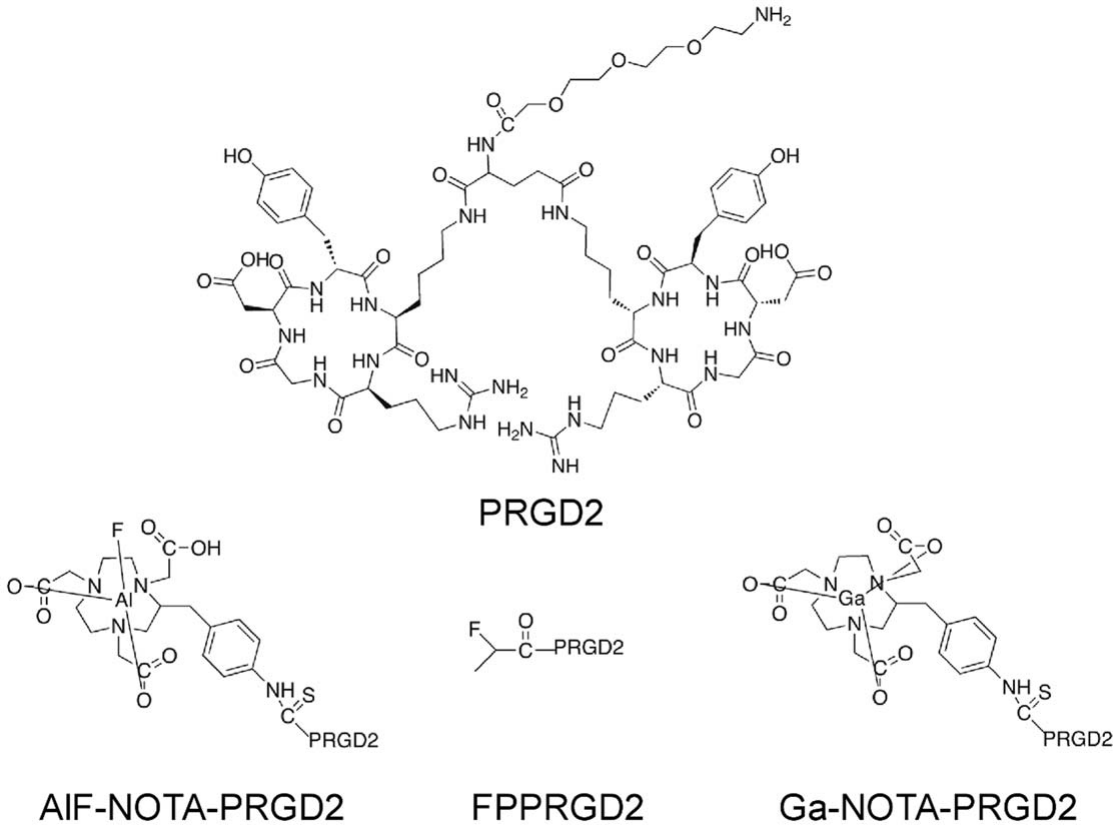
Chemical structures of three dimeric RGD peptides [^18^F]FPPRGD2, [^68^Ga]Ga-NOTA-PRGD2 and [^18^F]AlF-NOTA-PRGD2.

Similar to kinetic modeling using nonlinear least square method, the regular GA method also requires arterial blood sampling in order to obtain accurate input function. In some instances, e.g. receptor binding study, a reference region can be employed in place of arterial plasma input if it is devoid of the specific binding sites [30], [31]. The DV ratio (DVR) derived from reference tissue model generally provides better reproducibility than either the DV or the recepter parameter k_3_ [29]. The binding potential (Bp_ND_ = DVR-1) of tracer can be calculated on a voxel-by-voxel basis and used for voxel-wise comparison. Herein, we applied Logan graphical analysis with reference tissue to perform comparisons of pharmacokinetics between the more readily synthesized [^18^F]AlF-NOTA-PRGD2 and [^68^Ga]Ga-NOTA-PRGD2, and the previously established radioligand [^18^F]FPPRGD2 (**Figs. 1** **and** **2**).

**Figure 2:**
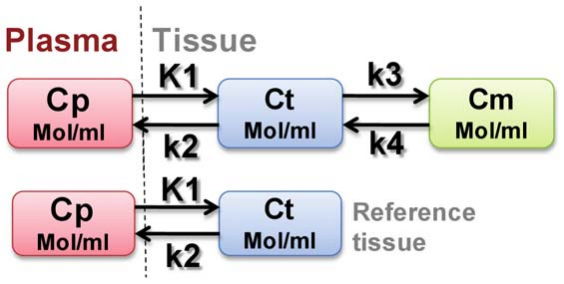
The three-compartment model describing RGD tracer kinetics in tumor and the two-compartment model describing RGD tracer kinetics in reference tissue. Cp represents tracer concentration in arterial blood plasma. Ct represents the free or non-specific binding of tracer in interstitial and intracellular space. Cm represents the portion of RGD tracer bound specifically to integrin. K_1_, k_2_, k_3_ and k_4_ are the transport and binding rates of the tracer. K1 [ml/g/min] reflects the perfusion rate into tissue. k_2_ [1/min] represents the clearance rate from plasma. k_3_ [1/min] is the specific binding rate and k_4_ [1/min] is the dissociation rate.

## Results

### Time-activity curves

**Figure 3:**
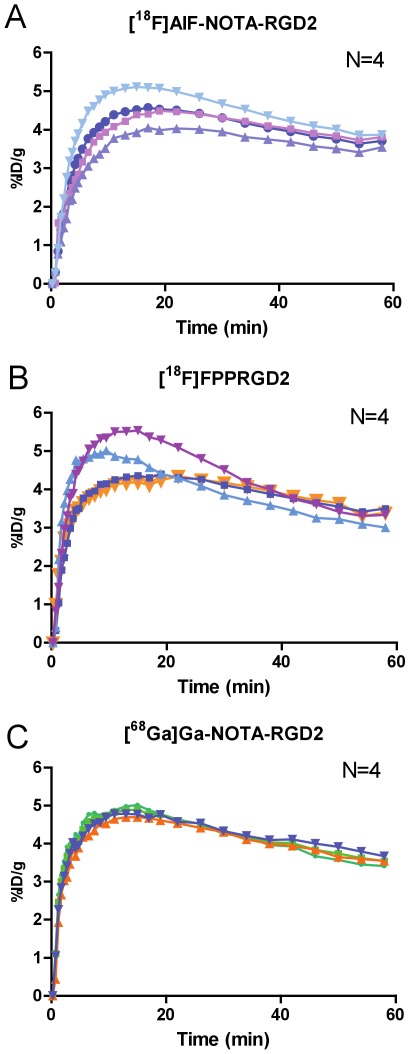
Tumor time-activity curves derived from 60-min dynamic PET scans of mice after administration of dimeric RGD peptide tracers. (**a**) [^18^F]AlF-NOTA-PRGD2, (**b**) [^18^F]FPPRGD2, and (**c**) [^68^Ga]Ga-NOTA-PRGD2 (n = 4/group).

**Figure 4:**
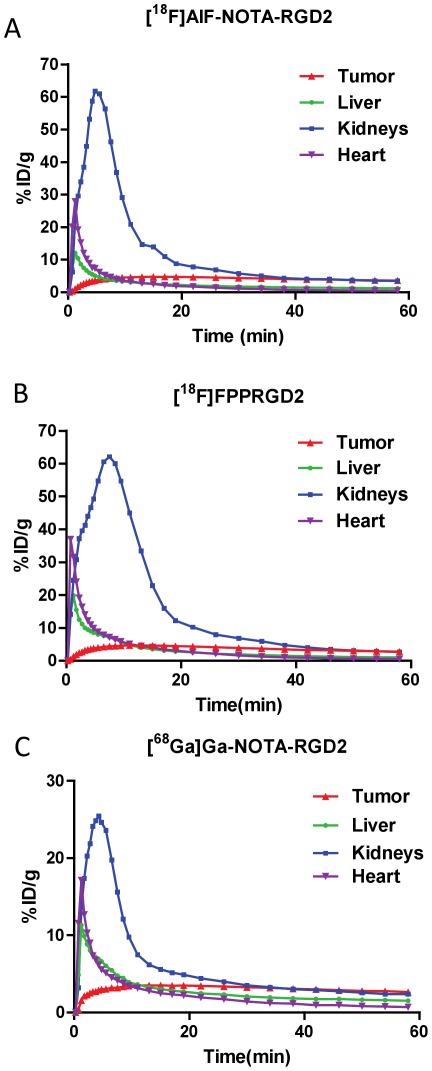
Representative time-activity curves of major organs (kidneys, heart, tumor and liver) derived from 60-min dynamic PET scans after administration of dimeric RGD peptide tracers. (**a**) [^18^F]AlF-NOTA-PRGD2, (**b**) [^18^F]FPPRGD2, and (**c**) [^68^Ga]Ga-NOTA-PRGD2 (n = 4/group).

Sixty-minute dynamic PET scans were performed to evaluate the pharmacokinetics and kinetic parameters for tumor targeting of [^18^F]AlF-NOTA-PRGD2, [^18^F]FPPRGD2 and [^68^Ga]Ga-NOTA-PRGD2. The time-activity curves were illustrated for the U87MG tumor (**Fig. 3**) and for the liver, kidneys and heart (**Fig. 4**).

For all three RGD probes, radioactivity accumulated rapidly in the tumor and remained high uptake throughout the dynamic scan period. All three tracers showed rapid and high initial kidney accumulation, reaching peak value at around 5 min after injection, followed by rapid clearance from the renal system over time. Other normal organs such as liver and heart showed a peak at early time points (<1 min) because of blood perfusion with high concentration of radioactivity. The uptakes in these regions dropped rapidly afterwards, which is consistent with a previous report [32].

### Quantification of static images

The quantification of three RGD tracers in tumor and main organs including liver, kidneys, and muscle were obtained from image at 1-h time point, which is the last frame of dynamic image series. The U87MG tumors were clearly identified with all three RGD tracers. The quantitative uptake values in tumor and other main organs were summarized in **Table 1**, shown as %ID/g. The tumor uptake of [^18^F]AlF-NOTA-PRGD2 was 3.45±0.18%ID/g (n = 4), which is significantly higher than that of either [^18^F]FPPRGD2 (2.91±0.35%ID/g, n = 4, p<0.05) or [^68^Ga]Ga-NOTA-PRGD2 (2.42±0.56%ID/g, p<0.05). Although all three groups showed relatively high kidney accumulation at 1-h time point, [^18^F]AlF-NOTA-PRGD2 showed much higher kidney uptake than the other two tracers (p<0.05). As shown in **Table 1**, [^18^F]AlF-NOTA-PRGD2 had significantly higher kidney uptake (4.67±1.08%ID/g) at 1-h time point than the other two RGD dimers ([^18^F]FPPRGD2: 2.78±0.58%ID/g, n = 4, p = 0.029; [^68^Ga]Ga-NOTA-PRGD2: 2.62±0.98%ID/g, p = 0.033). At the same time, all three tracers had relatively low liver uptake and no statistical difference was found among groups (p>0.05).

**Table 1:** Estimated parameter values from static images and kinetic modeling.

		Tumor	Kidneys	Muscle	Liver
Tissue uptake (%ID/g)	[^18^F]AlF-NOTA-PRGD2	3.45±0.18	4.67±1.08	0.53±0.11	1.21±0.54
	[^18^F]FPPRGD2	2.91±0.35	2.78±0.58	0.36±0.089	1.10±0.18
		(p = 0.033)	(p = 0.029)	(p = 0.110)	(p = 0.720)
	[^68^Ga]Ga-NOTA-PRGD2	2.45±0.56	2.62±0.98	0.60±0.11	1.36±0.28
		(p = 0.012)	(p = 0.033)	(p = 0.500)	(p = 0.590)
Volume of Distribution (V_T_)	[^18^F]AlF-NOTA-PRGD2	2.65±0.34	5.24±0.24	0.53±0.086	1.23±0.085
	[^18^F]FPPRGD2	2.35±0.22	4.10±0.27	0.44±0.071	0.97±0.14
		(p = 0.370)	(p = 0.002)	(p = 0.130)	(p = 0.015)
	[^68^Ga]Ga-NOTA-PRGD2	2.07±0.12	3.37±0.56	0.57±0.064	1.24±0.11
		(p = 0.110)	(p = 0.006)	(p = 0.440)	(p = 0.900)

P values indicate the significance of difference between [^18^F]AlF-NOTA-PRGD2 and the other two tracers respectively.

### Kinetic parameters estimation

**Figure 5:**
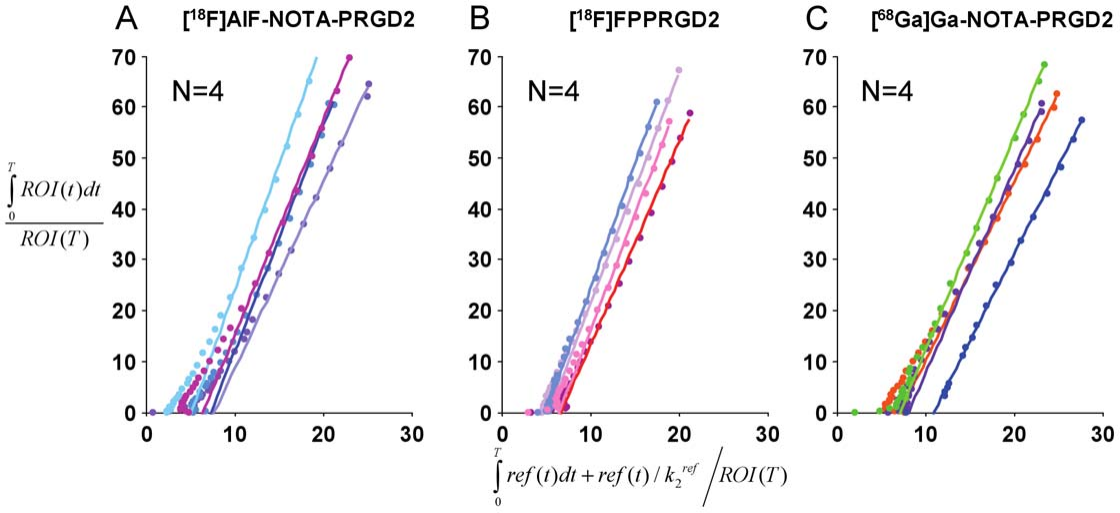
Logan graphical analysis fitting to 60-min dynamic microPET data, which showed excellent linearity of normalized integrated (Int) tumor activity vs. normalized integrated muscle tissue activity effective for time >30 min. Slopes of fits represent DVRs.

**Figure 6:**
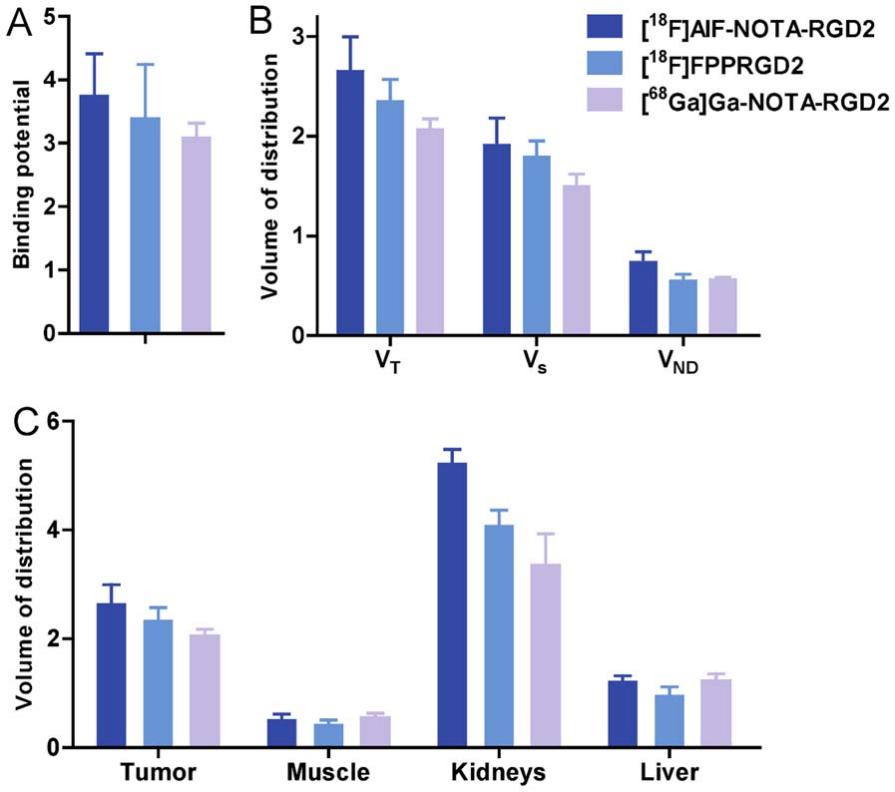
(**a**) Binding potential (Bp_ND_) of ^18^F-labeled RGD peptide tracers. (**b**) Volumes of distribution (V_T_) of ^18^F-labeled RGD peptide tracers. The Bp_ND_ was calculated as k_3_/k_4_ reflecting the binding affinity, and the volume of distribution (V_T_ = K_1_/k_2_(1+k_3_/k_4_)) reflects the tissue-to-plasma concentration ratio. V_T_ can be regarded as the sum of specific (V_S_ = K_1_·k_3_/(k_2_·k_4_)) and nonspecific (V_ND_ = K_1_/k_2_) distribution. (**c**) Volume of distribution of tumor, kidneys, muscle and liver.

Quantitative analysis and kinetic parameter estimation were performed by fitting the time-activity curves derived from 60-min dynamic PET data. To compare Bp_ND_ of all three RGD tracers for tumor targeting, Logan graphical analysis was performed using muscle as reference tissue. **Figure 5** shows the linear regression of normalized integration of tumor ROI and that of reference tissue (muscle) ROI. The best linear correlation was achieved when the exchange between tissue and plasma reached equilibrium (t*>30 min). The slope of the regression line was DVR and the Bp_ND_ (DVR-1) values of each tracer were computed. As shown in **Figure 6a**, [^18^F]AlF-NOTA-PRGD2 had slightly higher Bp_ND_ (3.75±0.65) in tumor than those of [^18^F]FPPRGD2 (3.39±0.84) and [^68^Ga]Ga-NOTA-PRGD2 (3.09±0.21), but the difference is not significant (p>0.05).

V_T_ was calculated according to Logan graphical analysis with image-derived input function. In the tumor region, V_T_ could be separated into non-displaceable and specific binding components to enable accurate assessment of the magnitude of specific binding of all three RGD compounds. **Figure 6b** plots the mean ± SD of the total V_T_, specific V_S_ and non-specific V_ND_ components of each tracer in tumor. V_S_ was found to be the dominant component of the total distribution volume in each group. [^18^F]AlF-NOTA-PRGD2 showed slightly higher V_S_ (1.92±0.26) than those of [^18^F]FPPRGD2 (1.79±0.15) and [^68^Ga]Ga-NOTA-PRGD2 (1.50±0.12), but no significant difference was found among them (p>0.05). Little difference in V_ND_ was found among different groups. To further compare the quantitative distribution of whole body organs, V_T_ of tumor, kidneys, muscle, and liver were also calculated and illustrated in **Figure 6c**. The mean and SD value of each macro-parameter were summarized in **Table 1**. Unlike the uptake calculated from static images, little difference was found in V_T_ among three RGD tracers in the tumor, liver and muscle. Meanwhile, V_T_ of [^18^F]AlF-NOTA-PRGD2 (5.24±0.24) in kidneys was higher than those of [^18^F]FPPRGD2 (4.10±0.27, n = 4, p = 0.002) and [^68^Ga]Ga-NOTA-PRGD2 (3.37±0.56, n = 4, p = 0.006), which correlated to the static analysis.

### Parametric mapping

**Figure 7:**
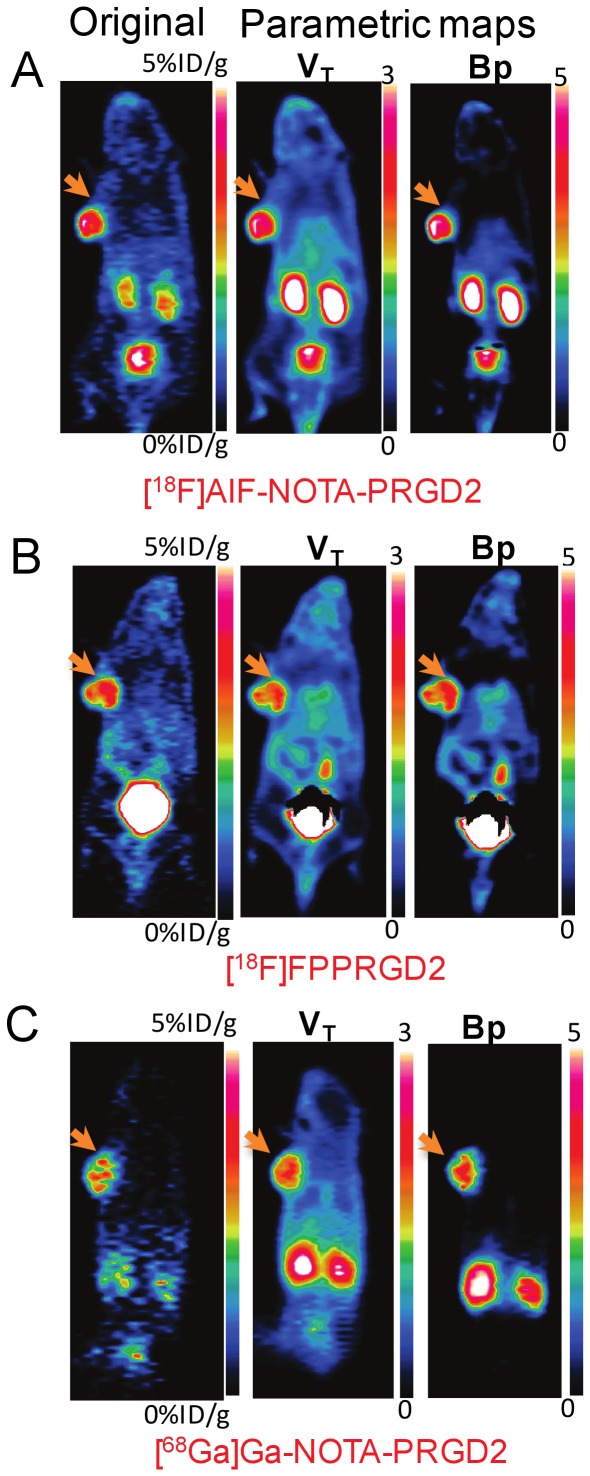
Representative original static PET images at 60 min (left), parametric maps of volume of distribution (middle) and binding potential (right) for RGD peptide tracers. (**a**) [^18^F]AlF-NOTA-PRGD2, (**b**) [^18^F]FPPRGD2, and (**c**) [^68^Ga]Ga-NOTA-PRGD2. The arrows point to tumors.

Parametric maps of V_T_ and Bp_ND_ were generated at voxel level by fitting the Logan graphical model to the time-activity curves of each voxel of the dynamic PET image series (**Fig. 7**). In Bp_ND_ maps, [^18^F]AlF-NOTA-PRGD2 showed comparable Bp_ND_ value in the tumor region with [^18^F]FPPRGD2 and [^68^Ga]Ga-NOTA-PRGD2. In the V_T_ parametric maps, all three tracers showed very similar volume of distributions in normal organs. Bp_ND_ maps also provided higher tumor-to-muscle contrast than the original static images for all three tracers. Compared with original static PET images at 60 min, parametric maps showed more comparable tumor region value, which is consistent with the quantification from static image analysis and kinetic parameter estimation.

## Discussion

Several groups, including ours, have pursued a straightforward and relatively high yield one-step RGD labeling procedure for pre-clinical and clinical applications. The preparation of [^18^F]AlF-NOTA-PRGD2 has been described in previous reports [25], [32]. In static image analysis, the quantification of tissue uptake, expressed as %ID/g has been well established and widely used in the quantification of molecular imaging. Other than the binding affinity of receptors, the uptake of a given radiotracer determined from static images at a particular time point can be affected not only by several microenvironment factors, such as the variance of blood perfusion, heterogeneous vascular permeability, but also by the pharmacokinetics in the body, for instance, the whole body blood circulation, clearance pattern from renal system, and tracer washout rate in the target tissue. Thus, kinetic modeling with dynamic imaging, which can provide tracer pharmacokinetic information and separate the actual specific binding component from total tracer uptake in tissue, will significantly facilitate the molecular probe pharmacokinetic evaluation. In this study, we evaluated the [^18^F]AlF-NOTA-PRGD2 using kinetic modeling in U87MG xenografts model and generated whole-body parametric maps with voxel level modeling for the first time. We compared the kinetic parameters with the well-established RGD dimer [^18^F]FPPRGD2 and another rapidly labeled RGD tracer ^68^Ga-NOTA-PRGD2.

Compared with static image analysis, dynamic PET imaging followed by kinetic estimation provides the time course of various organs and the quantitative characterization of tracer pharmacokinetics. Based on the compartment model, RGD tracer accumulation in the tumor region can be separated into three components: tracer in arterial plasma, non-specific or specific uptake, according to the homogeneous tracer concentration in plasma, interstitial space or tumor cells. The kinetic parameters (k_c_) reflect the exchange rates between compartments representing the intravascular, extravascular or interstitial, and intracellular transportation rate. By fitting the time-activity curves of dynamic PET data to the three-compartment model, non-specific and specific binding can be separated from the total tumor uptake and the actual specific binding affinity can be revealed. In the static image analysis, [^18^F]AlF-NOTA-PRGD2 (3.45±0.18%ID/g, n = 4) showed significantly higher tumor uptake than that of [^18^F]FPPRGD2 (2.91±0.35%ID/g, n = 4, p = 0.032) and [^68^Ga]Ga-NOTA-PRGD2 (2.42±0.56%ID/g, p = 0.012), which is consistent with the previous study [25]. However, the kinetic analysis demonstrated that [^18^F]AlF-NOTA-PRGD2 had comparable binding affinity with the other two RGD dimer peptides and no significant difference was found in either Bp_ND_ or V_T_ of the three RGD compounds (p>0.05). The parametric imaging also showed more comparable kinetic parameter values than the original static images in the tumor, which correlated well with the quantification of macro-parameters. Considering several factors contributing to static tumor uptake, the relatively higher value of [^18^F]AlF-NOTA-PRGD2 uptake may be a consequence of more non-specific accumulation and lower clearance rate from plasma. For [^18^F]FPPRGD2, the PEGylation improved the properties by reducing the renal retention of compounds [25]. According to the time-activity curves in kidneys, [^68^Ga]Ga-NOTA-PRGD2 showed faster renal clearance and correlated with the observation of fast washout in the plasma. Thus, kinetic modeling would eliminate the impact of non-specific binding that may be caused by blood flow, permeability variance and different interstitial fluid pressure. In this study, despite of the apparent difference in tumor uptake and clearance pattern, the actual specific binding in tumor region, which has been extrapolated from the kinetic modeling, appears to be similar.

Although no significant difference was found in kinetic parameters among all three RGD peptide tracers, [^18^F]AlF-NOTA-PRGD2 still showed a slightly higher Bp_ND_ and V_s_ in the tumor, and the lowest values were observed in the [^68^Ga]Ga-NOTA-PRGD2 group, as shown in **Figure 6a–b**. Because of the similar binding affinity and integrin expression level among all the tested animals, the difference between Bp_ND_ and specific volume of distribution may be a consequence of variance of specific activity ([^18^F]AlF-NOTA-PRGD2>[^18^F]FPPRGD2>[^68^Ga]Ga-NOTA-PRGD2 at the end of synthesis).

The pharmacokinetic analysis of RGD compounds has been conducted with ^64^Cu-DOTA-RGD [33], [^18^F]FPPRGD2 [34], and ^18^F-galacto-RGD [35] in preclinical or clinical studies. In our previous study, we used the Logan graphical analysis with reference tissue model to fit the dynamic time activity curves (TACs) for ^18^F-labeled RGD tracers [36]. These studies have implied that the RGD kinetics agrees with a reversible three-compartment model. Although Ferl *et al.* [33] conducted pharmacokinetic analysis of ^64^Cu-DOTA-RGD in preclinical models and demonstrated that a 2-tissue compartment, 4-parameter model with internalization was more appropriate to describe RGD tracer kinetics, the internalization of RGD tracer did not play a key role in the kinetic modeling especially for early time points (<60 min). Herein, we apply the kinetic analysis using Logan graphical analysis with reference tissue model based on the 3-compartment reversible model.

Considering the reasonable signal to noise ratio of dynamic PET data, we conducted model fitting and kinetic parameter estimation using linear regression. Similar to the GA with arterial plasma input function form, the reference region method is susceptible to noise. The main problem with GA is the bias in the estimated parameters due to noise [29]. Several multiple linear regression methods have been proposed to reduce such bias and improve the accuracy of parameter estimation, e.g. Ichise et. al. [37], Zhou et. al. [38], [39]. In the future, we will apply an average of values determined from some subset of these different methods to reduce the bias and variability, and may achieve superior results than applying any one of them [29] alone.

In conclusion, [^18^F]AlF-NOTA-PRGD2 and [^68^Ga]Ga-NOTA-PRGD2 have comparable pharmacokinetics and quantitative parameters compared to those of [^18^F]FPPRGD2. Despite the apparent difference in tumor uptake and clearance pattern, the actual specific binding extrapolated from the kinetic modeling appears to be comparable for all three RGD tracers. The satisfying performance in the whole body kinetic estimation and easy labeling procedure suggest that [^18^F]AlF-NOTA-PRGD2 is a promising alternative to [^18^F]FPPRGD2 for integrin targeting with PET.

## Materials and Methods

The ^68^Ge/^68^Ga generator was purchased from iThemba Labs (South Africa) and ^18^F-fluoride was obtained from the NIH cyclotron facility. [^18^F]FPPRGD2, [^68^Ga]Ga-NOTA-PRGD2 and [^18^F]AlF-NOTA-PRGD2 (**Fig. 1**) were prepared according to a published procedure [25].

### Preparation of animal tumor models

The U87MG human glioblastoma tumor model, which has been documented to express high level of integrin α_v_β_3_ [32], was selected for PET imaging. The U87MG cells obtained from ATCC (Manassas, VA) were cultured in DMEM medium supplemented with 10% fetal bovine serum (FBS), 100 IU/mL penicillin, and 100 µg/mL streptomycin (Invitrogen), and in a humidified atmosphere containing 5% CO_2_ at 37°C. The xenografted model was established by inoculation of 5×10^6^ cells into the left shoulder of each female athymic nude mouse at 5–6 weeks of age (Harlan Laboratories). Tumor growth was monitored by caliper measurements of perpendicular axes of the tumor three times a week after the tumors are palpable. The tumor volume was determined as the formula: V = a×(b^2^)/2, where a and b are the length and width of each tumor, respectively, in mm. The U87MG xenografted mice underwent PET imaging when the tumor volume reached about 300 mm^3^ (about 3 weeks after inoculation). This study was approved by the NIH Clinical Center Animal Care and Use Committee (ACUC). Moreover, all mice were maintained in a specific pathogen-free facility in accordance with the requirements of the ACUC.

### Dynamic PET imaging

Dynamic PET data acquisition was performed using an Inveon microPET scanner (Siemens Medical Solutions). With the assistance of the Inveon system's positioning laser, U87MG tumor-bearing mouse was placed with its tumor located at the center of field of view (FOV), where the highest imaging sensitivity can be achieved. Sixty-minute dynamic PET scans were performed after tail-vein injection of ∼3.7 MBq (100 µCi) of radiotracer ([^18^F]AlF-NOTA-PRGD2, [^18^F]FPPRGD2 or [^68^Ga]Ga-NOTA-PRGD2, n = 4/group) under isoflurane anesthesia. During the acquisition period, a thermostat-controlled thermal heater maintained the body temperature of mice. PET images were reconstructed with 2 iterations of 3-dimensional ordered-subsets expectation maximum (3D OSEM) with 14 subsets, followed by 18 iterations of maximum a posteriori (MAP) algorithm with a smoothing parameter of 0.1 (frame rates: 10×30 s, 5×60 s, 5×120 s and 10×240 s). No attenuation correction was performed in this study.

### ROI quantification and derivation of time-activity curves

In the dynamic PET image analysis, regions of interest (ROIs) were measured with the Inveon Research Workplace (IRW) 3.0 software. ROI was determined by manually superimposing the ellipsoid volume of interest (VOI) to the target tissue on the last frame of the entire 60-min dynamic image sequence. Then a threshold of 30% maximum was set to screen the voxels with lower values in the entire VOI because of possible tumor heterogeneity and shape irregularity. The time–activity curves were derived by superimposing the same VOI on each time frame of the entire 60-min dynamic image sequence and the value of each time point represents the overall concentration of radioactivity in the tissue. The activity concentrations were determined by the mean pixel intensity within each VOI, which were converted to µCi/ml using a calibration constant. Assuming the tissue density of 1 g/ml, the ROI activity was converted to µCi/g and normalized as percent injected dose per gram (%ID/g). The tissue uptake quantification of static scan at 60 min was determined from the last frame of dynamic images. The arterial blood input function was estimated by drawing a VOI in the region of left ventricle on the reconstructed PET image at the 0.5 min time point (the second frame of dynamic PET image series). The region of muscle contralateral to the tumor was selected as the reference tissue.

### Kinetic modeling and parameter estimation

Kinetic analysis of regional TACs was performed based on two-tissue (three-compartment) and one-tissue (two-compartment) model (Fig. 2). The three-compartment model consists of unmetabolized radiotracer in arterial blood plasma (Cp), free or non-specific binding tracer in interstitial and intracellular space (Ct), and tracer bound specifically to integrin (Cm). Both Ct and Cm occupy the same physical volume. The ROI(t) represents the sum of radioactivity from all compartments and includes the plasma volume fraction. Similarly, the two-compartment model describes RGD tracer kinetics using muscle as reference tissue and Ct_ref_ represents free (non-specific binding) tracer in the reference tissue (muscle) region. Generally speaking, kinetic parameters K_1_ [ml/g/min], k_2_ [1/min], k_3_ [1/min], k_4_ [1/min] represent the transport or binding rates of plasma perfusion into tissue, clearance from plasma, specific binding and dissociation, respectively.

Based on Logan plot shown in Eq. 1 [28], [33], the ratio between the integral of Cp(t) and the instantaneous value of ROI(t), and the ratio between the integral and the instantaneous value of ROI(t) become linearly related when the exchange between the target tissue and plasma reaches an equilibrium (t>t*).
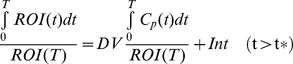
(1)DV denotes the distribution volume and can be easily calculated from the linear regression. DV is a measure of the capacity of tissue to bind a particular tracer and can be regarded as the sum of specific (V_S_) and nonspecific distribution (V_ND_).
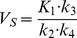
(2)

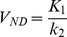
(3)Total volume of distribution is defined in equation 4.
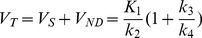
(4)K_1_, k_2_, k_3_, and k_4_ are calculated by linear fitting to 60-min dynamic PET data [34].

In the original Logan plot estimation, arterial blood input function is required. Unfortunately, blood sampling faces technical challenges and brings radiation exposure to researchers. A reformulation of the Logan analysis, which uses a reference region, provides the possibility to estimate the kinetic parameters without arterial blood sampling [31]. We select muscle as the reference tissue because of its negligible integrin expression and have the relationship between muscle and plasma expressed in equation 5.
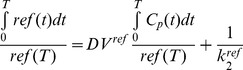
(5)Thus, the normalized integral of activity in the tumor versus the normalized integral of activity in the muscle becomes linear according to Eq.6:
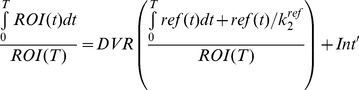
(6)The ratio of integrated tumor uptake and tumor uptake was set as the y-axis. The ratio of integrated reference tissue uptake and tumor uptake was set as the x-axis in Logan plot. The slope of the linear portion of the Logan plot is distribution volume ratio (DVR). Binding potential (Bp_ND_ = k_3_/k_4_), a macro-parameter reflecting the binding affinity *in vivo*, could be derived from DVR (Bp_ND_ = DVR-1).

### Parametric map estimation

Voxel-wise parametric mapping was generated for whole body image using Logan plot. Logan graphical analysis with input function was performed to calculate V_T_ at voxel level using Eq. 1. Reference tissue model was applied for Bp_ND_ map according to Eq. 5 and 6 [34], [35].

### Statistical Analysis

Quantitative kinetic parameters determined from dynamic PET data were expressed as mean ± SD. Differences between either parameters derived from static images and kinetic analysis or kinetic parameters among all three RGD groups were evaluated using unpaired Student *t* test. P values less than 0.05 were considered statistically significant.
